# Therapeutic Potential of Stem Cell-Derived Exosomes in Skin Wound Healing

**DOI:** 10.3390/biomimetics10080546

**Published:** 2025-08-20

**Authors:** ChanBee Jo, Yun Ji Choi, Tae-Jin Lee

**Affiliations:** Department of Medical Biotechnology, Division of Biomedical Convergence, College of Biomedical Science, Kangwon National University, Chuncheon-si 24341, Republic of Korea; chanbi723@kangwon.ac.kr (C.J.); cyj0887@naver.com (Y.J.C.)

**Keywords:** adipose-derived stem cells, chronic wounds, exosomes, induced pluripotent stem cells, mesenchymal stem cells, skin wound regeneration

## Abstract

Chronic skin wounds are difficult to heal or nonhealing. These wounds may become infected and progress to tissue necrosis, potentially leading to limb amputation, sepsis, reduced quality of life, depression, economic burden on the healthcare system, and social isolation. Several clinical strategies, including negative pressure wound therapy, antibiotic-based infection control, and wound debridement, have been developed to treat skin wounds. However, these approaches primarily target local wound conditions and offer only short-term relief, not achieving sustained functional regeneration. Stem cell-based therapy has emerged as an alternative therapeutic method for skin wound treatment owing to its ability to suppress inflammation, stimulate angiogenesis, and promote cellular proliferation. However, the low post-transplantation survival rate of stem cells remains a major limitation. Exosomes, nanosized extracellular vesicles, transport proteins, lipids, mRNAs, and miRNAs and mediate regenerative functions, including anti-inflammatory effects, angiogenesis promotion, and extracellular matrix remodeling. Stem cell-derived exosomes (SC-Exos) offer several advantages over their parent cells, including greater stability, lower immunogenicity, absence of tumorigenic risks, and ease of storage and distribution. These attributes render SC-Exos particularly attractive for cell-free regenerative therapies. In this review, we introduce exosomes derived from various types of stem cells and explore their therapeutic applications in skin wound regeneration.

## 1. Introduction

Chronic wounds refer to wounds that do not progress through the normal stages of wound healing or do not heal within three months [1]. These wounds, often referred to as hard-to-heal or non-healing wounds, are characterized by disruptions or delays in the normal wound healing phases: hemostasis, inflammation, proliferation, and remodeling [2]. Common types of chronic wounds include diabetic foot ulcers, pressure ulcers, and venous ulcers, all of which are increasing in prevalence globally owing to aging populations and the increase in incidence of chronic diseases [3]. Multiple factors, including peripheral vascular disease, chronic inflammation, infection, tissue ischemia, sustained pressure, and diabetic microangiopathy, contribute to the development of chronic wounds [2]. Collectively, these conditions lead to blood flow reduction, oxygen deprivation, and impaired immune cell function, disrupting tissue homeostasis at the wound site [2]. In addition to simple tissue defects, chronic wounds result in infection and tissue necrosis, potentially leading to limb amputation, sepsis, reduced quality of life, depression, economic burden on the healthcare system, and social isolation [4,5,6]. Notably, approximately 20% of patients with diabetic foot ulcers undergo amputation, with a five-year post-amputation mortality rate of 50% [7]. Therefore, there is an urgent need for therapeutic strategies for chronic wounds that extend beyond superficial wound coverage and promote functional tissue regeneration [7].

**Figure 1:**
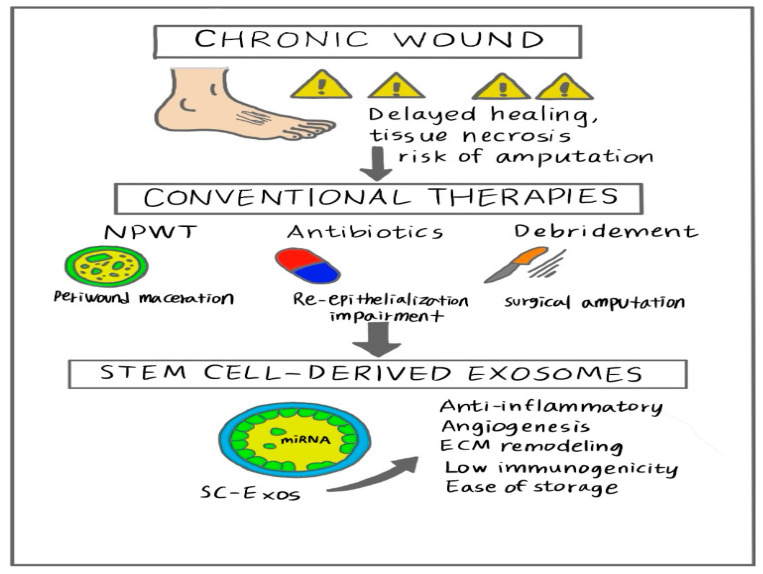
Comparison of conventional therapies and stem cell-derived exosomes in chronic wound treatment. While conventional approaches target local symptoms, SC-Exos offer a regenerative solution by modulating inflammation, promoting angiogenesis, and supporting tissue remodeling.

To address the clinical challenges associated with chronic wounds, various conventional therapeutic strategies, such as negative pressure wound therapy (NPWT), antibiotic-based infection control, and wound debridement, have been developed (Figure 1) [8]. NPWT is a noninvasive technique that applies controlled negative pressure at the wound site under [5]. NPWT minimizes skin wound size, removes exudates, promotes blood flow, and stimulates granulation tissue formation and angiogenesis, all of which promote wound healing [5,9]. However, NPWT may cause minor adverse effects, such as pain during dressing changes, tissue necrosis, tissue erosion, and periwound maceration [10,11,12]. In chronic wounds, bacterial colonization can occur without affecting the healing process [13]. However, when bacterial levels reach a critical threshold, wound healing is impaired, and infections may spread to surrounding tissues [13]. Although local antibiotic treatment can support infection control, antibiotics do not significantly alter the overall microbial diversity or abundance in patients with diabetic foot ulcers. Antibiotics may even enhance the virulence of certain pathogenic bacteria and impair re-epithelialization [14,15]. Wound debridement, which involves removing non-viable tissue, can be performed using non-mechanical (autolytic, enzymatic) or mechanical (sharp/surgical, ultrasound, wet-to-dry debridement, biosurgery/maggot debridement therapy, and aqueous high-pressure lavage) methods [16]. Debridement prevents the growth of pathological organisms and reduces inflammation during the early stages of the healing process [16]. However, in some cases, infection cannot be fully controlled, and surgical amputation of damaged tissue becomes essential [5]. Despite the widespread use of these therapies, they primarily target local wound conditions and provide short-term relief; however, they do not promote sustained, functional regeneration, highlighting the need for a paradigm shift in chronic wound treatment.

In recent years, stem cell-based therapies have emerged as a promising alternative method for wound healing and tissue regeneration [17]. Among these, mesenchymal stem cells (MSCs), in particular, have the ability to suppress inflammation, stimulate angiogenesis, and promote cellular proliferation, which could enhance wound healing [17]. However, the therapeutic efficacy of MSCs is limited by their low post-transplantation survival [18]. In a study using NOD mice, luciferase-labeled syngeneic MSCs were rapidly cleared within seven days post-transplantation, primarily due to apoptosis and immune rejection by macrophages and natural killer cells [18]. Consequently, attention has shifted to the paracrine mechanisms of MSCs, particularly their secreted exosomes, as a cell-free therapeutic alternative. Tutuianu et al. (2021) demonstrated that MSC-derived exosomes modulate and enhance the biological activities of keratinocytes and fibroblasts and promote angiogenesis in vitro, thereby promoting wound healing without the risks associated with cell transplantation [19]. These findings suggest that exosomes can replicate the regenerative effects of MSCs via paracrine signaling, offering a safer and more standardized treatment strategy [19].

Exosomes are nano-sized extracellular vesicles (30–150 nm) that are secreted from all cell types. Exosomes consist of a phospholipid bilayer that carries bioactive molecules, such as proteins, lipids, mRNAs, and miRNAs [20,21]. These vesicles facilitate intercellular communication and contribute to tissue regeneration by exerting anti-inflammatory effects, promoting angiogenesis, and supporting extracellular matrix remodeling [20,22]. Stem cell-derived exosomes (SC-Exos) present several advantages over their parent cells, including greater stability, lower immunogenicity, absence of tumorigenic risk, and ease of storage and distribution (Figure 1) [22]. These characteristics make SC-Exos particularly attractive for cell-free regenerative therapy [23,24].

In this review, we aimed to explore the therapeutic potential of stem cell-derived exosomes in the treatment of chronic skin wounds, offering a comprehensive overview of their mechanisms, benefits, and clinical translational prospects in regenerative medicine. While many previous studies have focused on exosomes derived from a single stem cell source, this review offers a broader analysis by encompassing a variety of stem cell types, including MSCs, adipose-derived stem cells (ADSCs), induced pluripotent stem cells (iPSCs), human umbilical cord MSCs (HucMSCs), and hair follicle mesenchymal stem cells (HF-MSCs). By systematically discussing the biological mechanisms, therapeutic efficacy, and clinical applicability of exosomes derived from each cell type, this review aims to present an integrated perspective on the distinct characteristics and potential of various stem cell-based exosome therapies. Therefore, it offers a more comprehensive and differentiated discussion compared to the existing literature.

## 2. Molecular Mechanisms of Skin Wound Healing Process

**Figure 2:**
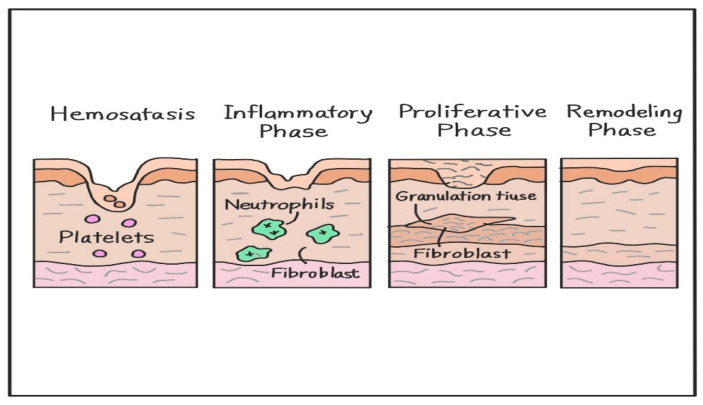
The wound healing process progresses through four main stages: hemostasis, inflammation, proliferation, and remodeling. Each phase is defined by distinct cellular activities, such as platelet aggregation, immune cell infiltration, granulation tissue formation, and collagen remodeling.

Wound healing is a complex, multiphase biological process involving hemostasis, inflammation, proliferation, and remodeling; this process is tightly regulated through cellular interactions, molecular signaling pathways, and extracellular matrix (ECM) dynamics (Figure 2) [17,25,26]. Following injury, the hemostatic response initiates clot formation to prevent blood loss [27]. During the inflammatory phase, immune cells, such as neutrophils and monocytes, infiltrate the wound to clear debris and pathogens [28]. CD4^+^ and CD8^+^ lymphocytes also accumulate in significant numbers within skin wounds, peaking on days 5–10 and 7–10 post-injury, respectively [29]. In CD4- or CD8-deficient mice, lymphocyte depletion alters the timing and extent of inflammatory cell infiltration and cytokine production; however, wound closure is not impaired [29]. Once the wound is closed, the remodeling phase can persist for several months. During this time, the overall cell population declines, inflammation resolves, neovascular networks are reorganized, and type III collagen is gradually replaced by type I collagen to restore tissue strength and functionality [30]. These cellular dynamics support the transition from inflammation to repair, enabling successful wound resolution without impairing tissue closure [28,29].

### 2.1. Hemostasis

Following vascular injury, circulating platelets rapidly adhere to the exposed subendothelial matrix, activating platelets and initiating a sequence of events that lead to clot formation and vascular sealing (via a platelet plug) [27]. Concurrently, the coagulation cascade is activated, converting fibrinogen into fibrin, stabilizing the clot [27]. Simultaneously, activated platelets released chemokines, such as platelet-derived growth factor (PDGF) and transforming growth factor-β (TGF-β), which initiate the recruitment of inflammatory cells and fibroblasts to the wound site [31]. Thus, homeostasis restores vascular integrity and sets the stage for inflammation and tissue proliferation [31].

### 2.2. Inflammatory Phase

During this phase, neutrophils infiltrate injured tissues to phagocytose pathogens and damaged tissue. Next, monocyte-derived macrophages initially adopt a pro-inflammatory M1 phenotype and later transition into the anti-inflammatory M2 phenotype to promote tissue repair [28]. In a murine sterile wound model, circulating monocytes differentiated into Ly6C^hi^ inflammatory macrophages, which then transitioned into Ly6C^lo^–MerTK^+^ repair macrophages around day 14, a process that reflects the resolution of inflammation and the onset of tissue repair [28]. Exosomal miRNAs, such as miR-146a and miR-223, play key roles in this transition by inhibiting NF-κB signaling and suppressing NLRP3 inflammasome activation, respectively [32,33]. Macrophages also release cytokines, such as TNF-α, IL-1β, and IL-6, to amplify the immune response and clear debris [17,27]. Preconditioned MSC-derived exosomes further enhance anti-inflammatory polarization via let-7b signaling [34]. Maintaining the balance between pro- and anti-inflammatory signals is critical, as dysregulation may lead to chronic wounds or fibrosis [35].

### 2.3. Proliferative Phase

This phase is characterized by re-epithelialization, angiogenesis, and fibroblast activation [35]. Keratinocytes migrate across the wound bed to restore the epidermis, while endothelial cells sprout new capillaries under the influence of vascular endothelial growth factor (VEGF) and fibroblast growth factor 2 (FGF-2) [35]. Fibroblasts infiltrate the wound bed and secrete type III collagen and fibronectin, forming a granulation tissue scaffold [35]. Additionally, TGF-β1 activates fibroblasts to synthesize ECM [35]. Exosomes from MSCs and ADSCs enhance fibroblast proliferation and migration by delivering miRNAs, such as miR-21, miR-29a, and miR-200b, which modulate the PI3K/Akt and TGF-β/Smad3 signaling pathways [36,37,38]. Furthermore, exosomal miR-126 and miR-132 promote angiogenesis in wounded tissues by stimulating endothelial cell migration and tube formation in the wound microenvironment [32,33].

### 2.4. Remodeling Phase

During the remodeling phase, myofibroblasts and excess endothelial cells undergo apoptosis, thereby reducing tissue cellularity [34]. The initial type III collagen is gradually replaced with type I collagen, restoring the tensile strength of the regenerated tissue [35]. ECM turnover is mediated by the dynamic interplay between matrix metalloproteinases (MMPs) and tissue inhibitors (TIMPs); specifically, the MMP-9/TIMP-1 ratio in wound fluid is significantly lower in healed pressure ulcers compared to those with poor or intermediate healing outcomes [39]. Exosomes enriched in miR-29a and TGF-β1 promote organized collagen deposition and minimize scarring [31,38]. In this final phase, the skin barrier is restored, and tissue elasticity is improved [35].

## 3. Exosomes

**Figure 3:**
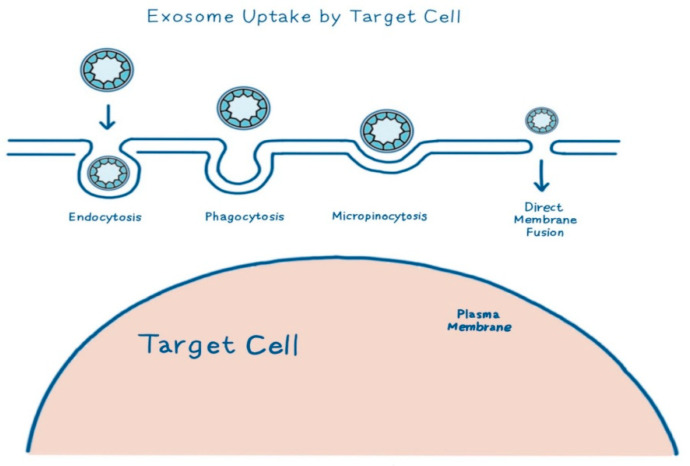
Exosomes interact with target cells via various uptake mechanisms—endocytosis, phagocytosis, micropinocytosis, and direct membrane fusion—enabling the delivery of therapeutic miRNAs, proteins, and lipids into the recipient cytoplasm.

Exosomes are extracellular nanovesicles ranging from 30 to 150 nm, derived from the endosomal pathway. These nanovesicles function as intercellular messengers, transporting molecules such as proteins, miRNAs, and lipids [19] that modulate the behavior of recipient cells. Their biogenesis begins with the inward budding of the endosomal membrane, forming multivesicular bodies (MVBs). Upon fusion with the plasma membrane, intraluminal vesicles (ILVs) are released as mature exosomes [40]. This process is tightly regulated by the ESCRT complex (TSG101, VPS4, and Alix) and, in some cases, by tetraspanins, such as CD9, CD63, and CD81, which also serve as key markers of exosome identification [36,41]. Exosomes exhibit considerable heterogeneity, influenced by the cell source, physiological conditions, and biogenesis route; these factors affect therapeutic potential and targeting specificity [42]. The molecules that are transported by exosomes reflect their cellular origin and physiological state and typically comprise nucleic acids (e.g., miR-21, miR-29a, and miR-150-5p), proteins (e.g., TGF-β1 and VEGF), lipids (e.g., sphingomyelin, cholesterol, and phosphatidylserine), and enzymes [33,41,43]. These molecules actively influence the wound healing process. For instance, exosomal miR-21-3p enhances fibroblast proliferation and collagen production via PI3K/Akt signaling activation through PTEN suppression [44], while miR-29a downregulates TGF-β2/Smad3 signaling to suppress fibrosis and scar formation [38]. Lipids, such as sphingomyelin and cholesterol, abundant in exosome membranes, increase membrane fluidity and facilitate the fusion of exosomes and recipient cells [45]. Exosome uptake by target cells occurs through several mechanisms, including endocytosis, phagocytosis, micropinocytosis, and direct membrane fusion, depending on the exosome surface proteins and target cell type (Figure 3) [42]. Together, the integrated action of these miRNAs, proteins, and lipid components confers several therapeutic advantages: low immunogenicity, efficient tissue penetration, and customizable molecular payload [42]. In addition to their intrinsic therapeutic features, exosomes can be engineered or chemically modified to enhance therapeutic efficacy. For example, miR-146a-loaded exosomes embedded in silk fibroin patches demonstrated superior anti-inflammatory and collagen remodeling effects in wound healing models [46]. Storage stability is critical for wound healing [46]. Notably, Sc-Exos exhibit high stability during long-term storage, retaining over 90% bioactivity even after lyophilization or freezing at −80 °C [46].

## 4. Stem Cell-Derived Exosome for Skin Wound Treatment

Sc-Exos have emerged as promising therapeutic agents for skin wound healing [47]. Sc-Exos can be administered via intravenous and subcutaneous injections [23]. The delivered Sc-Exos exert therapeutic effects via inflammation inhibition, immune response modulation, wound closure and angiogenesis promotion, cell proliferation and migration enhancement, cell–cell communication activation in fibroblasts and keratinocytes, and scar formation reduction by regulating the proportion of collagen [47,48,49]. Among stem cells, MSCs, ADSCs, and iPSCs are the most extensively studied exosome sources for wound healing [41,50,51]. These exosomes demonstrate distinct mechanisms and therapeutic profiles based on their origin [41,50,51]. Herein, we describe skin wound regeneration using MSC-, ADSC-, and iPSC-derived exosomes. In addition, we introduced other types of stem cell-derived exosomes for skin wound treatment.

### 4.1. Mesenchymal Stem Cell-Derived Exosome

**Figure 4:**
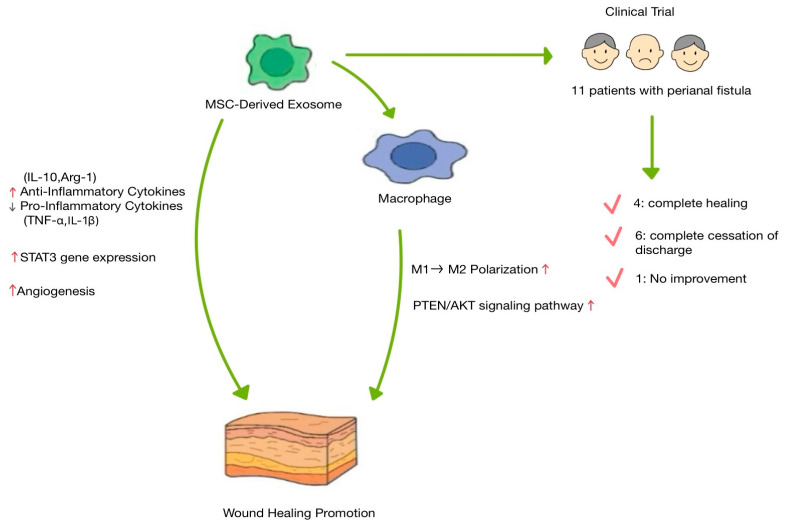
Representative mechanisms of MSC-derived exosomes in cutaneous wound healing.

MSC-derived exosomes enhance skin wound regeneration by modulating the inflammatory response, promoting fibroblast proliferation, and stimulating angiogenesis (Figure 4). Various inflammatory cells and factors are involved in this inflammatory phase [42]. While important for regeneration, long-term inflammation results in excessive wound scarring or chronic refractory wounds [42]. Macrophages play an important role in this process. Initially, macrophages adopt a pro-inflammatory M1 phenotype and later transition into a regenerative pro-inflammatory M2 phenotype [28]. If the M1-M2 transition is not induced, wounds progress into chronic refractory wounds; therefore, the M1-M2 transition is critical in wound regeneration [52]. MSC-Exos have the potential to induce M2 polarization [53,54]. Exosomes isolated from bone marrow-derived mesenchymal stem cells (BMMSCs), human jawbone marrow-derived mesenchymal stem cells (JMMSCs), and melatonin-stimulated MSCs have the potential to induce M2 polarization and accelerate skin wound regeneration in mouse models [53,54]. Exosomes derived from both BMMSCs and JMMSCs enhanced wound healing in vivo, accompanied by increased expression of M2 macrophage markers, such as Arg-1 and CD206 [53]. Melatonin, a hormone that is widely distributed in the body, enhances fat-derived exosome transfer to macrophages, M2 transformation, and inhibition of fat inflammation [55]. Exosomes derived from melatonin-stimulated MSCs enhance the secretion of anti-inflammatory cytokines, such as IL-10 and Arg-1, while reducing the expression of pro-inflammatory cytokines, such as IL-1β and TNF-α, in macrophages in vitro [54]. Also, in vivo, exosomes derived from melatonin-treated MSCs improve M2 polarization by activation of the PTEN/AKT signaling pathway [54].

MSC-mediated endogenous repair or tissue regenerative mechanisms are based on the secretion of soluble or paracrine factors [17,56,57,58]. Treatment of normal or diabetic wound fibroblasts with MSC-derived exosomes enhanced proliferation and migration as well as tube formation by endothelial cells [51]. Exosomes derived from melatonin-stimulated MSCs improve angiogenesis and collagen synthesis in skin wounds, and MSC-Exo enhance the expression of STAT3 genes that control cellular processes, such as cellular proliferation, migration, and angiogenesis [51,54].

Based on these findings, MSC-derived exosomes were used in clinical trials to treat perianal fistula [59]. Perianal fistula is an immune-mediated chronic recurrent disease with an abnormal connection between the epithelium and the skin of the anal canal [60]. Owing to their immunomodulatory properties, stem cells were highlighted as alternative treatments for perianal fistula [61,62]. In a phase I clinical trial, exosomes derived from placenta-derived MSCs were administered to 11 patients with complex perianal fistulas who met the inclusion criteria [59]. After treatment, four patients showed complete healing [59]; the external orifices were completely epithelialized. In six patients, discharge stopped completely, while in four patients, the discharge volume was reduced [59]. Only one patient showed no change in fistula discharge [59]. Although the sample size was small, these results indicate that MSC-derived exosomes could be utilized as a safer and more novel therapeutic option for the treatment of patients with perianal fistula compared to MSCs.

### 4.2. Adipose-Derived Stem Cell-Derived Exosome

**Figure 5:**
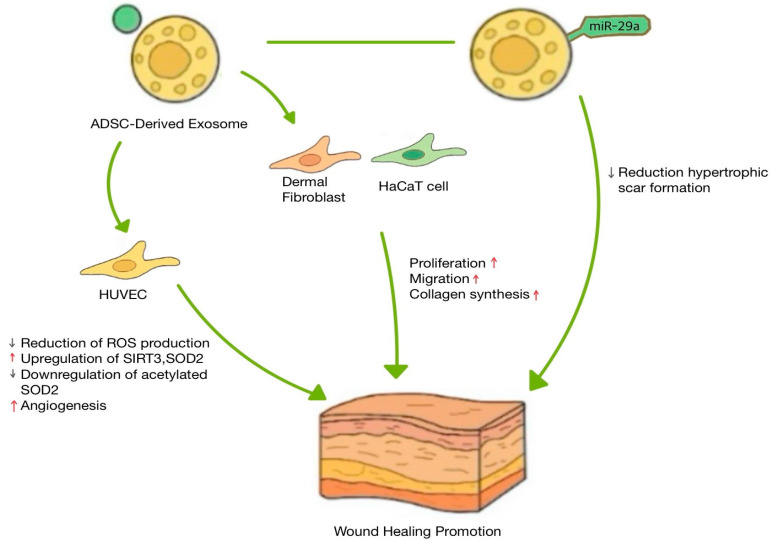
Representative mechanisms of ADSC-derived exosomes in cutaneous wound healing.

ADSCs are widely used adult stem cells that enhance wound healing and reduce scar formation [63,64,65]. Similar to MSCs, the therapeutic functions of ADSCs mainly involve exosomes, paracrine cytokines, and other acellular bioactive derivatives [66]. Therefore, ADSC-derived exosomes have been widely used for skin wound regeneration. ADSC-derived exosomes improved skin wound regeneration by enhancing fibroblast proliferation and angiogenesis (Figure 5). ADSC-derived exosomes are internalized by fibroblasts, enhancing cellular proliferation, migration, and collagen synthesis in vitro, resulting in enhanced wound regeneration in vivo [23]. Interestingly, intravenously administered, these exosomes showed faster wound closure than the locally injected group [23]. It was confirmed that fibroblast migration and collagen deposition in the exosome-treated group were elevated [23].

ADSC-derived exosomes to wound regeneration is the improvement of angiogenesis through the regulation of oxidative stress [67]. Exosomes derived from normal human subcutaneous adipose tissue stem cells delivered to a skin wound promoted angiogenesis and wound closure [67]. When human umbilical vein endothelial cells (HUVECs) proliferate, migrate, and undergo angiogenesis, they are elevated [67]. Interestingly, ADSC-derived exosomes reduced ROS production, protected mitochondrial function, and promoted SIRT3 expression [67]. SIRT3 is a mitochondrial deacetylase that improves oxidative stress, regulates mitochondrial function, and reduces ROS production in endothelial cells [68]. When SIRT3 expression was reduced, VEGF expression was inhibited, antioxidant capacity was impaired, and wound healing [69]. Therefore, ADSC-derived exosomes can enhance angiogenesis by reducing ROS production, thereby improving wound regeneration.

hADSC-derived exosomes have the ability to deliver miRNA cargo that promotes wound regeneration [38] (Figure 5). During the wound healing process, excessive collagen deposition, along with increased fibroblast proliferation and activation, contributes to the development of hypertrophic scars and keloids [70]. These scars often result in pain, itching, and aesthetic concerns, significantly negatively impacting patient quality of life [38]. Therefore, the prevention of scar formation during wound healing is considered a crucial objective in clinical trial treatment strategies [71]. miR-29a inhibits fibroblast proliferation, migration, and collagen deposition after thermal skin injury and improves denatured dermis repair [72,73]. Additionally, miR-29a downregulation is closely associated with keloid formation due to excessive fibroblast proliferation [38]. miR-29a expression is downregulated in mouse scar tissues and human hypertrophic scar fibroblasts [38]. And overexpressed miR-29a exosomes in human ADSCs inhibit the proliferation and migration of human hypertrophic scar fibroblasts, enhancing wound healing, reducing morphological changes, and inhibiting epidermal overgrowth [38]. In a molecular mechanism study, it was shown that miR-29a overexpression activates the TGF-β2/Smad3 signaling pathway [38].

Although exosomes offer promising therapeutic effects, their application in wound healing is still associated with some limitations [74]. For example, exosomes have a rapid clearance rate and short half-life in vivo and cannot accumulate at the wound site for a long time at high concentrations [75]. Therefore, the need for biocompatible carriers that can load exosomes, maintain their bioactivity, and induce sustained delivery in clinical applications has increased [74]. Biomaterials can further enhance the wound healing efficacy of ADSC-derived exosomes. Hydrogels offer a suitable microenvironment for wound healing and are promising drug carriers [76,77]. ECM, a direct survival environment of cells, is one of the best sources of hydrogels that mimic the in vivo environment, and ECM-based hydrogels improve site-appropriate remodeling response in vivo [78,79,80,81]. Therefore, a thermosensitive ECM-based hydrogel incorporating ADSC-derived exosomes was fabricated to enhance skin wound regeneration [74]. ADSC-derived exosome-loaded ECM hydrogels were fabricated by mixing the ECM solution with ADSC-derived exosomes [74]. ADSC-derived exosomes were sustained-released from hydrogel, enhanced the proliferation of HaCaT cells and HUVECs, the migration of HaCaT cells, and tube formation by HUVECs [74]. ADSC-Exos also significantly enhance fibroblast migration and collagen synthesis and wound closure in diabetic ulcer models [74]. It was also demonstrated that the exosome-loaded hydrogel enhances wound regeneration in both normal and diabetic wound models [74].

### 4.3. Induced Pluripotent Stem Cell-Derived Exosome

**Figure 6:**
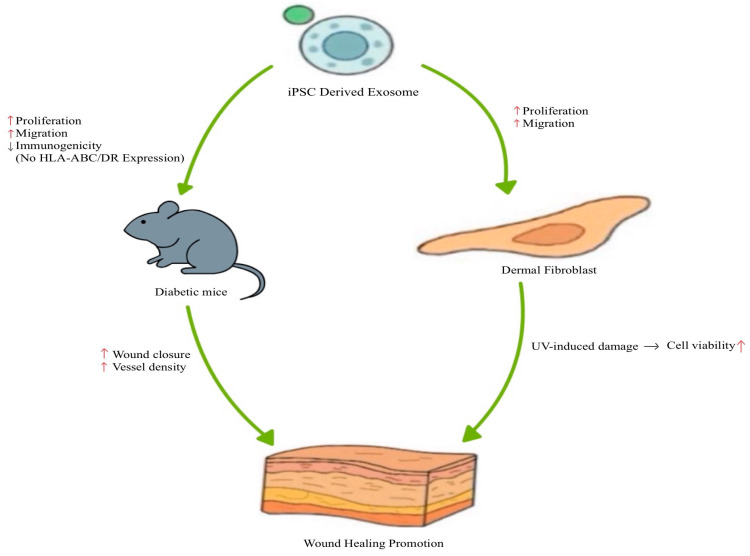
Representative mechanisms of iPSC-derived exosomes in cutaneous wound healing.

In recent years, owing to their unique properties, such as reduced immune response, differentiation into various cell types, and avoidance of ethical issues, iPSCs have emerged as a promising source for cell therapy [82]. However, iPSC-based cell therapies are associated with several disadvantages, including pulmonary infarction and tumor formation [83]. Therefore, conditioned media from cell cultures have been highlighted as a novel method to overcome these disadvantages and have shown effectiveness in promoting wound regeneration [84,85,86]. These findings suggest that exosomes or macrovesicles present in conditioned media are promising and important contributors to wound healing [83]. iPSC-derived exosomes (iPSC-Exos) represent an emerging class of cell-free therapeutics, offering potential advantages for standardization and large-scale production [82]. Therefore, iPSC-derived exosomes have been investigated for their application in skin wound regeneration [83,87] (Figure 6). Exosomes from the iPS cell line (201B7) did not express HLA-ABC and HLA-DR, indicating low immunogenicity [83]. iPSC-derived exosomes significantly enhanced fibroblast (isolated from diabetic mice) proliferation and migration [83]. Furthermore, in vivo wound healing experiments in diabetic mice demonstrated that iPSC-derived exosomes accelerated wound closure and increased vessel density by day 7 post-treatment [83]. iPSC-derived exosomes were used to treat skin aging [87]. There are two types of skin aging: intrinsic aging, which is genetically determined, and extrinsic aging, which is caused by external factors, such as air pollution, smoking, unbalanced nutrition, and UV exposure [87]. UV exposure damages the skin and induces irregular pigmentation, sallowness, dryness, roughness, premalignant lesions, and skin cancer [88]. Fibroblasts, the primary cell type in the dermis that synthesize procollagen and elastic fibers, lose their ability to proliferate and synthesize collagen [89,90]. This reduces the skin regeneration capacity [88]. Recently researchers demonstrated that iPSC-conditioned medium stimulates fibroblast proliferation and migration [91]. Based on these findings, the researchers hypothesized that the exosomes present in iPSC-conditioned medium play a critical role in mediating these effects [87]. The isolated exosomes enhanced proliferation and migration of human dermal fibroblasts [87]. Additionally, iPSC-derived exosomes protected human dermal fibroblasts from UV-induced damage [87]. iPSC-derived exosomes increased collagen type I expression and reduced matrix-degrading enzymes, MMP-1 and MMP-3 [87]. Finally, iPSC-derived exosomes reversed senescence-related gene expressions of human dermal fibroblasts [87]. These results indicate that iPSC-derived exosomes enhance fibroblast proliferation and migration, as well as confer protection against UV-induced cellular damage [87].

### 4.4. Other Type of Stem Cell-Derived Exosome

**Figure 7:**
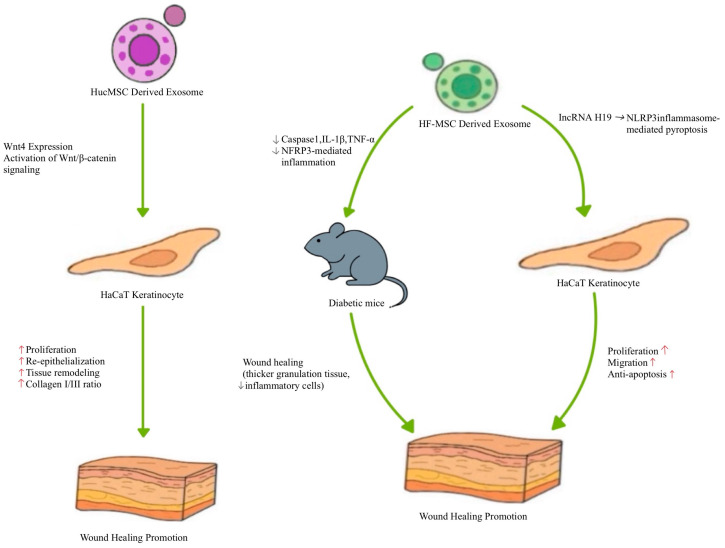
Representative mechanisms of HucMSCs and HF-MSC-derived exosomes in cutaneous wound healing.

Human umbilical cord MSCs (HucMSCs) are inexhaustible and can be obtained at a low cost without invasive procedures [92,93]. HucMSCs are primitive MSCs with high plasticity and developmental flexibility [94]. Therefore, HucMSCs are an attractive source of cells for regeneration. HucMSCs-derived exosomes reduce liver fibrosis and improve renal injury repair [95,96]. These results indicate that, like MSC, HucMSC-derived exosomes may be the key factors in HucMSC-mediated skin wound regeneration [97]. Exosomes were isolated from HucMSCs and were administered to a rat model of deep second-degree burn injury to evaluate their therapeutic effects [97] (Figure 7). After two weeks, re-epithelialization was enhanced in groups treated with HucMSC- or HucMSC-derived exosomes, indicating improved tissue remodeling [97]. Additionally, the ratio of collagen I to collagen III gene expression was higher in HucMSCs- or HucMSC-derived exosome-treated groups, indicating improved tissue remodeling [97]. In an in vitro burn injury model, treatment with HucMSCs-derived exosomes prevented heat stress-induced apoptosis in HaCAT cells and dermal fibroblasts [97]. Interestingly, after heat stress, these HucMSCs-derived exosome-treated HaCAT cells and dermal fibroblasts showed enhanced proliferation [97]. Molecular analysis revealed that HucMSCs-derived exosomes express elevated levels of Wnt4, which activated wnt/β-catenin signaling, ultimately contributing to improved wound healing outcomes [97]. These findings suggest that HucMSCs-derived exosomes protect against heat stress-induced apoptosis and promote skin wound regeneration via Wnt4-mediated wnt/β-catenin signaling activation.

A recent study highlighted the therapeutic potential of exosomes derived from HF-MSC (HF-MSC-Exos) in the treatment of diabetic skin wounds [98] (Figure 7). The authors found that these exosomes were enriched with long non-coding RNA (lncRNA) H19, which promotes fibroblast proliferation and migration while preventing apoptosis [98,99]. Based on these findings, the researchers hypothesized that lncRNA H19 in HF-MSC-Exos could regulate human fibroblast activity and enhance wound healing in diabetic mouse skin [98]. When HF-MSC-Exos were used to treat HaCaT cells cultured in high glucose conditions, NLRP3 inflammasome-mediated pyroptosis was prevented [98]. Mechanistic studies using HF-MSC-Exos overexpressing or silencing H19 revealed that lncRNA H19 promoted proliferation, migration, and protection against apoptosis via NLRP3 pyroptosis signaling pathway modulation [98]. Furthermore, in a diabetic mouse skin wound model, delivery of H19-overexpressing HF-MSC-Exos improved wound healing, as evidenced by the presence of thicker granulation tissue and fewer inflammatory cells in the wound [98]. H19-overexpressing exosomes downregulated caspase1, IL-1β, and TNF-α, indicating suppression of NFRP3-mediated inflammation [98]. In summary, HF-MSC-Exos hold significant potential as a novel therapeutic approach for enhancing skin wound healing in diabetic skin wounds [98].

## 5. Challenges and Future Perspectives

**Figure 8:**
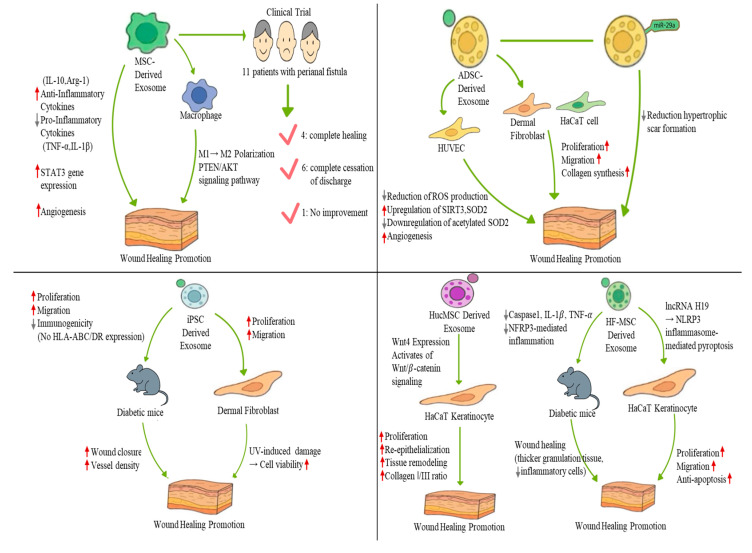
Representative mechanisms of stem cell-derived exosomes in cutaneous wound healing.

**Figure 9:**
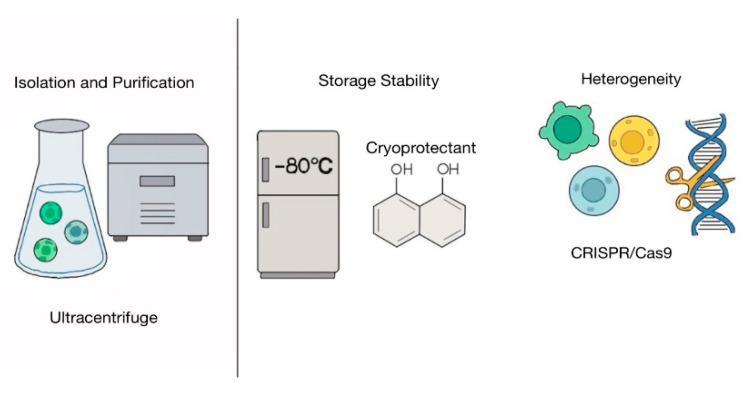
Key challenges in the clinical translation of stem cell-derived exosomes.

In this review, we found that exosomes can be readily obtained from various stem cells, including MSCs, ADSCs, iPSCs, HucMSCs, and HF-MSCs. These exosomes demonstrated their ability to enhance skin wound regeneration in vitro and in vivo, and in clinical applications (Table 1). Based on these findings, stem cell-derived exosomes hold significant potential as a cell-free therapeutic strategy for skin wound healing (Figure 8). However, despite their promising potential, several challenges limit their widespread clinical application. One major hurdle is the isolation and purification of exosomes from cells (Figure 9) [100]. Exosomes are a subtype of extracellular vesicles secreted by cells [101]. Extracellular vesicles are broadly categorized into three subtypes: exosomes, macrovesicles, and apoptotic bodies [100]. These subtypes share overlapping physicochemical properties, including size and density [101], which makes it difficult to isolate exosomes with high purity. Moreover, exosomal surface marker expression can vary depending on cell type and even under different conditions within the same cell [102]. This further complicates the development of standardized isolation methods. Common techniques for exosome isolation, such as ultracentrifugation, ultrafiltration, size-exclusion chromatography, precipitation, and immunoaffinity capture, present significant technical challenges and may not yield highly pure or consistent exosome preparations [100]. Since high-quality and well-characterized exosomes are essential for therapeutic use, there is an urgent need to develop more reliable, efficient, and standardized isolation and characterization techniques. Recently tangential flow filtration (TFF), which concentrates and filters particles using a cross-flow filtration principle, has emerged as a promising alternative, offering significantly higher yields of small extracellular vesicles compared to ultracentrifugation and is considered more suitable for large-scale and clinical-grade exosome production [103]. Storage stability is another critical challenge in the clinical application of exosome-based therapies [104]. Although exosomes are promising alternative therapeutics as agents for cell-free therapy, considered promising therapeutic agents for cell-free therapy, their biological nature makes long-term storage difficult [104]. Currently, the most widely accepted method for exosome preservation is ultra-low temperature storage at −80 °C [104]. However, the optimal storage conditions can vary depending on the exosome source and the experimental protocols used [104]. Importantly, repeated freeze–thaw cycles or prolonged storage at −80 °C can lead to a substantial reduction in exosome particle count and a significant loss of functional components, including key miRNAs [105,106]. Therefore, developing effective preservation strategies that maintain the structural and functional integrity of exosomes is essential for their clinical application [104]. One promising approach to address this limitation involves the use of cryoprotective agents, such as trehalose [107,108,109,110]. Trehalose has been identified as an effective and less cytotoxic cryoprotectant compared to traditional agents like DMSO, making it more suitable for human therapeutic use [107,108,109,110]. Notably, a cryoprotectant formulation combining trehalose with low-molecular-weight hyaluronic acid has been shown to preserve both the structural and functional properties of exosomes for up to six months at room temperature following lyophilization [110]. Various preservation methods such as freezing or freeze-drying with antifreeze and spray drying have been explored [104]. Despite these efforts, research is still ongoing to identify optimal cryoprotective agents and protocols tailored to the unique characteristics of different types of exosomes.

Exosomes are a heterogeneous group of cell-derived membranous structures [111]. Their cargo composition and functional properties vary between cell types and within the same cell under different physiological or pathological conditions [111]. This inherent heterogeneity makes it difficult to ensure consistent therapeutic effects and poses challenges in reproducing therapeutic outcomes across different patients [111]. To enable the clinical application of exosomes, further studies are needed to characterize their molecular and functional diversity and identify specific markers or signatures that correlate with distinct exosomal functions [111]. To address exosomal heterogeneity and enhance therapeutic consistency, various methods were explored [112]. For example, through genetic modification using CRISPR/Cas9, miR-29b-enriched exosomes have been successfully engineered to improve therapeutic outcomes [112]. Additionally, as discussed in earlier sections, the use of advanced materials and targeted drug delivery systems has shown promise in enhancing the efficacy and specificity of exosome-based therapies [74].

Although the potential for inducing immune responses remains a concern in the clinical application of SC-Exos, recent studies have proposed various strategies to address this limitation [113,114]. Given that HLA gene polymorphisms can influence the immunomodulatory functions and therapeutic efficacy of exosomes, future strategies incorporating HLA genotyping are expected to enable more precise and safer exosome-based treatments [115]. In particular, EVs expressing HLA-G have demonstrated the ability to modulate recipient immune response. Therefore, a personalized approach based on individual HLA profiles may contribute to minimizing immune rejection [115]. From this perspective, designing exosomes that match the recipient’s HLA genotype could represent a critical advancement in the development of SC-Exos as personalized immunotherapeutic agents [115].

Amid ongoing advancements in biological and technological fields, the development of automated production systems compliant with Good Manufacturing Practice (GMP) standards has significantly progressed, aiming to improve efficiency and consistency of quality control. This includes the use of high-quality materials, cells, and cell culture environments; advanced manufacturing techniques; and trained personnel operating under highly controlled and strictly monitored conditions for clinical application [116,117]. One notable innovation is the EXODUS system, which utilizes vibrational flow and dual-membrane filtration technologies. This system has been reported to collect more exosomes than other isolation methods [118]. It produces exosomes with a uniform particle size distribution centered around 100 nm (ranging from 50 to 200 nm) and uses a chromatography-like multistep purification mechanism capable of removing more than 99% of protein contaminants [118]. Furthermore, it supports high-throughput processing, enabling more efficient exosome recovery compared to conventional techniques, and has demonstrated the highest particle yield among various isolation platforms [118].

Additionally, purification and quality control procedures are essential to ensure that the final product satisfies the highest standards before use [116]. Advancements across multiple domains, including production technologies, storage strategies, immune safety, and automated manufacturing systems, are essential for successful clinical translation of SC-Exos [119,120]. Moreover, integration with emerging technologies such as three-dimensional printing and artificial intelligence (AI)-based diagnostics is expected to accelerate the application of SC-Exos in precision medicine [121,122]. Three-dimensional printing technology enables the fabrication of customized scaffolds that facilitate the sustained and controlled release of exosomes while preserving the designed structural integrity to support effective tissue regeneration through immunomodulation and enhancement of osteogenic and angiogenic activities [122]. AI-powered diagnostic systems, exemplified by platforms like ChatExosome, utilize deep learning models to analyze spectroscopic profiles of exosomes, enabling accurate and non-invasive detection of hepatocellular carcinoma and enhancing the interpretability of classification outcomes to support clinical decision-making [121]. These technological innovations are anticipated to significantly enhance the precision of personalized therapies and introduce a new treatment paradigm in the field of skin regeneration using SC-Exos [121,122,123].

Although there are currently no standardized biomarkers in clinical practice to predict patient responsiveness to stem cell-derived exosome (SC-Exos) therapies, several promising candidates have recently emerged. For instance, elevated activation of β-catenin and c-myc has been observed at the non-healing margins of chronic wounds and can be detected via immunohistochemistry, suggesting their potential utility as tissue-based prognostic markers [124]. Additionally, a high MMP-9 to TIMP-1 ratio in wound fluid has been associated with delayed healing, indicating that this ratio may serve as a practical predictor of therapeutic response, particularly in pressure ulcers [125]. Advances in single-cell RNA sequencing (scRNA-seq) have also enabled the identification of senescence-related genes, such as Rpl11 and Ccl2, in immune cell subsets within wound fluid—molecular features that are strongly linked to chronic or refractory wound states [126]. Moreover, in patients with chronic venous ulcers, elevated plasma levels of IL-15 and RANTES have shown a significant correlation with wound healing outcomes, underscoring their potential as serum-based prognostic biomarkers [127]. While these findings represent substantial progress toward the clinical application of molecular and genetic indicators, large-scale validation and standardization efforts remain essential for their routine implementation in clinical settings.

Biomaterial-based exosome delivery systems had emerged as a promising approach to preserve the structural stability and bioactivity of exosomes. Biomaterials can provide sustained, controlled, and function-specific release during exosome administration and can enhance the half-life of exosomes, thereby improving their therapeutic potentials [128,129,130]. In previous research by Lee et al., exosomes derived from adipose tissue–derived mesenchymal stem cells (ASC-EXOs) were incorporated into a hyaluronic acid (HA) hydrogel, which enhanced wound closure rates by approximately 10% compared to HA treatment alone [131]. This combination also promoted re-epithelialization and type III collagen deposition, contributing to scarless tissue repair [131]. Moreover, pH-responsive biomaterials allow for selective exosome release in the acidic microenvironment of chronic wounds [132]. Ghauri et al. developed a chitosan/guar gum/polyvinylpyrrolidone (PVP) ternary blended hydrogel that exhibited differential swelling behavior across a pH range of 1.2 to 10, with the highest swelling observed under acidic conditions, suggesting its potential as a controlled drug delivery platform [132]. Taken together, these exosome–biomaterial composite systems offer enhanced protection against exosome degradation and prolong their biological activity, thereby presenting a next-generation, cell-free therapeutic strategy for targeted skin wound repair. However, the therapeutic efficacy of exosome-based treatments can vary depending on the severity and type of wound; it is challenging and needs more investigation to determine the most effective exosome delivery strategy using biomaterials.

The clinical application of stem cell-derived exosomes (SC-Exos) for skin regeneration has raised several safety-related concerns. A case of skin necrosis was reported following intradermal injection of lyophilized exosomes, with histopathological analysis revealing necrotic keratinocytes, leukocytoclastic vasculitis, and eccrine gland necrosis [133]. In a toxicological assessment conducted by Ha et al. (2020), exosomes derived from adipose-derived stem cells were found to be safe for topical use; however, invasive administration routes, such as intradermal injection, have not received clinical approval [134]. A recent meta-analysis also highlighted substantial interindividual variability in treatment outcomes at days 7 and 14 post-exosome application and emphasized the ongoing lack of long-term safety data [135]. Therefore, the therapeutic use of SC-Exos in skin regeneration should proceed with caution until standardized protocols for production, characterization, and clinical evaluation are firmly established.

## 6. Conclusions

Chronic skin wounds pose several serious challenges, including infection, tissue necrosis, potential limb amputation, sepsis, reduced quality of life, depression, increased economic burden on the healthcare system, and social isolation. While stem cell-based therapies have shown promise in promoting wound healing, they are associated with significant clinical challenges and limitations. Exosomes, which are key molecular-induced paracrine factors secreted by stem cells, have emerged as a promising alternative therapeutic strategy owing to their regenerative abilities. This review has highlighted that exosomes from MSCs, ADSCs, iPSCs, HucMSCs, and HF-MSCs contribute to skin wound regeneration via modulation of inflammatory responses, promotion of cell proliferation and migration, enhancement of angiogenesis, and inhibition of cell apoptosis. Despite these promising results, several challenges remain for clinical application of SC-Exos, including issues related to isolation, storage, heterogeneity, immune compatibility, and large-scale manufacturing. However, ongoing advancements in biotechnology and biomedical engineering are steadily addressing these limitations. Therefore, Sc-Exo-based therapies represent a promising next-generation strategy for skin regeneration and wound healing, with the potential to transform current clinical practices.

## Figures and Tables

**Table 1:** Summary of experimental studies evaluating the therapeutic effects of stem cell-derived exosomes in wound healing.

Stem Cell Source	Study Types	Key Mechanisms	Therapeutic Outcome	Reference
MSC -derived exosomes	In vitro and in vivo	- Induces M2 macrophage polarization (↑ Arg-1, CD206) - Impaired exosome secretion leads to reduced M2 polarization and delayed wound healing	- Promoted wound regeneration in skin wound models - Increased M2 markers and accelerated wound healing	[53]
		- Inhibits M1 polarization and promotes M2 polarization of macrophages - Enhances IL-10, Arg-1 secretion; reduces IL-1β, TNF-α expression - Activates the PTEN/AKT signaling pathway	- Improved M2 polarization and wound healing in vivo - Promoted angiogenesis and collagen synthesis in skin wounds	[54]
		- Promotes fibroblast proliferation and migration - Enhances tube formation by endothelial cells - Increases STAT3 gene expression	- Improved cellular functions related to proliferation, migration, and angiogenesis in normal and diabetic fibroblasts	[51]
	Clinical trial	- Immunomodulatory effects of MSC-exosomes utilized for chronic inflammation-related condition	- Among 11 patients with complex perianal fistula: → Four showed complete healing → Six had complete cessation of discharge → One had no improvement	[59]
ADSC -derived exosomes	In vitro and in vivo	- Exosomes internalized by fibroblasts - Promotes fibroblast proliferation, migration, and collagen synthesis	- Accelerated wound healing in a mouse model - Enhanced collagen deposition - IV delivery is more effective than local injection	[23]
		- Reduces ROS production in HUVECs - Enhances mitochondrial function - Upregulates SIRT3 and SOD2, downregulates acetylated SOD2	- Promoted angiogenesis and wound closure in diabetic wounds - Protected endothelial cells under high-glucose conditions	[67]
		- miR-29a inhibits fibroblast proliferation, migration, and collagen deposition - Activates the TGF-β2/Smad3 signaling pathway	- Reduced hypertrophic scar formation - Improved wound healing and dermis repair in thermal injury model	[38]
		- Sustained exosome release (95% in 72 h) from thermosensitive ECM hydrogel - Promotes fibroblast migration, collagen synthesis, and tube formation	- 92% wound closure in diabetic ulcers - Enhanced wound regeneration in both diabetic and normal wound models	[74]
iPSC -derived exosomes	In vitro and in vivo	- Enhances fibroblast proliferation and migration (from diabetic mice) - Exhibits low immunogenicity (no HLA-ABC/DR expression)	- Accelerated wound closure in diabetic mice - Increased vessel density by day 7 post-treatment	[83]
		- Enhances fibroblast proliferation and migration - Proposed to mediate anti-aging effects	- Improved cell viability following UV-induced damage	[87]
hUCMSC, derived exosomes	In vitro and in vivo	- Wnt4 expression activates Wnt/β-catenin signaling - Protects HaCaT cells and fibroblasts from heat stress-induced apoptosis	- Enhanced re-epithelialization and tissue remodeling in second-degree burn wounds - Increased collagen I/III ratio - Improved fibroblast proliferation post-heat stress	[97]
HF-MSC derived exosomes	In vitro and in vivo	- lncRNA H19 modulates NLRP3 inflammasome-mediated pyroptosis - Promotes fibroblast proliferation, migration, and anti-apoptosis	- Improved wound healing in diabetic mouse skin (thicker granulation tissue, fewer inflammatory cells) - Downregulated caspase-1, IL-1β, TNF-α (reduced inflammation)	[98]

## Abbreviations

The following abbreviations are used in this manuscript:

SC-Exos	Stem cell-derived exosomes
NPWT	Negative pressure wound therapy
MSCs	Mesenchymal stem cells
ECM	Extracellular matrix
PGDF	Platelet-derived growth factor
TGF-β	Transforming growth factor-β
VEGF	Vascular endothelial growth factor
FGF-2	Fibroblast growth factor 2
ADSCs	Adipose-derived stem cells
MMPs	Matrix metalloproteinases
TIMPs	Tissue inhibitors
MVBs	Multivesicular bodies
ILVs	Intraluminal vesicles
iPSCs	Pluripotent stem cells
BMMSCs	Bone marrow-derived mesenchymal stem cells
JMMSCs	Human jawbone marrow-derived mesenchymal stem cells
HUVECs	Human umbilical vein endothelial cells
iPSC-Exos	iPSC-derived exosomes
HucMSCs	Human umbilical cord MSCs
lncRNA	Long non-coding RNA
HF-MSC-Exos	Hair follicle mesenchymal stem cells
EV	Extracellular vesicles
TFF	Tangential flow filtration
GMP	Good Manufacturing Practice
